# Traumatic Paralysis of the Facial Nerve

**Published:** 1871-07

**Authors:** Alfred E. Walker


### TRAUMATIC PARALYSIS OF THE FACIAL NERVE. RECOVERY.

BY ALFRED E. WALKER, M.D.

Last November, a girl about twelve years of age came to the Davis Dispensary to have a tumor attended to, which showed itself just below the tempero-maxillary articulation of the right side. Dr. Sherman tried the administration of absorbents for a while, but without any effect, and then he decided to remove it by an operation. This was accomplished, though the tumor was found to be much more extensive than was anticipated, extending in between the carotids, and being attached to the parotid gland back of the ramus of the jaw.

The next day the child was quite comfortable, but complained of numbness on the right side of the face. Twenty-four hours after the operation she commenced vomiting; twelve hours later the wound was dressed, and considerable fluid escaped; another twelve hours and the vomiting ceased; in the course of the next sixteen hours she had several convulsions. This brought her to the sixtieth hour after the operation, and subsequently there were no bad symptoms at all. The wound suppurated and healed, cicatrization being complete at the end of three weeks.

Now, as to the paralysis, it appeared the first time she was seen after the tumor was excised, and it exhibited all the usual symptoms of paralysis of the facial nerve, which need not be enumerated here. There was also anæsthesia for a space three-quarters of an inch broad, immediately in front of the incision, reaching from just under the ear to near the angle of the jaw.

On the 16th of January, that is, five weeks after the operation, the use of electricity was commenced with Kidder's machine. At this time the features had fallen downwards on the affected side, and there was a little ectropion of the lower eyelid. The girl holding one electrode in her hand, if the other was applied behind the cicatrix, no effect was produced; but if it was applied over the parotid gland in front of the cicatrix most of the facial muscles were set twitching. After this the battery was used every second day, the muscles moving more and more. On the first of February, it was found that pain could be excited in the surface in front of the cicatrix, but perception was not definite, as appeared from the girl's failure to tell correctly how many points touched her, though the compasses were separated by one-third of an inch. On the 14th of Feb., her sister came with her, and, in answer to questions, said that the patient had laughed a little on the right side for two weeks. She was accordingly provoked to smile, and straightway demonstrated her capacity for bilateral mirthfulness. The sponge applied behind the cicatrix now caused contractions in the facial muscles.

But two sets of muscles remained deficient in power or in innervation, namely, the orbicularis palpebrarum to raise the lower lid, and the depressors of the angle of the mouth and of the lower lip. If reflex action, or what Carpenter might call "consensual" action, was excited by pushing the finger roughly into the inner canthus, the eye would close tightly, but not in response to mere volition. And as to the mouth, no motion at all could be excited in the depressors, while the levators were gaining strength day by day, and finally threatened to give to the right side the appearance of a permanent grin. On the 11th of March, a galvanic current from a sixteen-cell battery was applied to the chin, and this caused the depressors to act. It was repeated on several occasions until the patient's visits became so irregular that treatment had to be discontinued.

Among the points of interest in this case we might, perhaps, include the vomiting, which commenced twenty-four hours after the operation. It may be attributable to the effects of the ether; but, on the other hand, as it did not begin until after a whole day it may have been occasioned by irritation of the glosso-pharyngeal nerve, by the contents of the wound, or by the thickening of the parts.

It will be noticed that on the 14th of February, the patient was said to have been laughing on the right side for two weeks. This would make such voluntary action of the muscles begin at about the same time that the space in front of the cicatrix was first found to be sensitive; and it is probable that if the sponge had then been applied to the back of the cicatrix the re-established continuity of the nerve would have been demonstrated. Though it is possible that the renewed sensibility was due to the growth of new nerves from adjacent branches, for the sensations perceived were very indefinite,—not accurately localized.

It seems to have been one month after external cicatrization was complete that the nerve recovered its conductivity. The depressors of the mouth had been quiescent three months when the galvanic current set them in motion.

Perhaps it is worth mentioning that the girl was running about in the open air during the coldest weather last winter, without any covering over the eye which she could not close, but that the eye never showed any sign of irritation, except watering a little.

